# Natural products for migraine: Data-mining analyses of Chinese Medicine classical literature

**DOI:** 10.3389/fphar.2022.995559

**Published:** 2022-10-28

**Authors:** Claire Shuiqing Zhang, Shaohua Lyu, Anthony Lin Zhang, Xinfeng Guo, Jingbo Sun, Chuanjian Lu, Xiaodong Luo, Charlie Changli Xue

**Affiliations:** ^1^ The China-Australia International Research Centre for Chinese Medicine, STEM College, RMIT University, Melbourne, VIC, Australia; ^2^ The Second Affiliated Hospital of Guangzhou University of Chinese Medicine, Guangdong Provincial Hospital of Chinese Medicine and Guangdong Provincial Academy of Chinese Medical Sciences, Guangzhou, China

**Keywords:** migraine, Chinese herbal medicine, classical literature, data-mining, association rule, Apriori algorithm

## Abstract

**Background:** Treatment effect of current pharmacotherapies for migraine is unsatisfying. Discovering new anti-migraine natural products and nutraceuticals from large collections of Chinese medicine classical literature may assist to address this gap.

**Methods:** We conducted a comprehensive search in the *Encyclopedia of Traditional Chinese Medicine* (version 5.0) to obtain migraine-related citations, then screened and scored these citations to identify clinical management of migraine using oral herbal medicine in history. Information of formulae, herbs and symptoms were further extracted. After standardisation, these data were analysed using frequency analysis and the Apriori algorithm. Anti-migraine effects and mechanisms of actions of the main herbs and formula were summarised.

**Results:** Among 614 eligible citations, the most frequently used formula was *chuan xiong cha tiao san* (CXCTS), and the most frequently used herb was *chuan xiong*. Dietary medicinal herbs including *gan cao*, *bai zhi*, *bo he*, *tian ma* and *sheng jiang* were identified. Strong associations were constructed among the herb ingredients of CXCTS formula. Symptoms of chronic duration and unilateral headache were closely related with herbs of *chuan xiong*, *gan cao*, *fang feng*, *qiang huo* and *cha*. Symptoms of vomiting and nausea were specifically related to herbs of *sheng jiang* and *ban xia*.

**Conclusion:** The herb ingredients of CXCTS which presented anti-migraine effects with reliable evidence of anti-migraine actions can be selected as potential drug discovery candidates, while dietary medicinal herbs including *sheng jiang*, *bo he*, *cha*, *bai zhi*, *tian ma*, and *gan cao* can be further explored as nutraceuticals for migraine.

## 1 Introduction

Migraine is a chronic condition manifesting as recurrent, unilateral, pulsing headache attacks, and associated with symptoms of nausea, vomiting, photophobia and phonophobia (Headache Classification Committee of the International Headache Society (IHS), 2018). According to a systematic analysis on the Global Burden of Disease Study, migraine affected 1.04 billion people and caused 45.1 million years of life lived with disability globally in 2016 (Stovner et al., 2018).

Pharmacological treatments such as calcium channel blockers, beta-blockers, and calcitonin gene-related peptide (CGRP) antagonists, have been recommended for managing migraine (Pringsheim et al., 2012; Silberstein et al., 2012). However, these treatments are associated with unsatisfied therapeutic effects, high costs and unwanted side effects, leading patients to seek complementary and alternative medicine (CAM) for their migraine relief (Wells et al., 2011), and encouraging scientists to continue new drug discovery (Schytz et al., 2017; Tauchen, 2020).

Natural products are valuable sources for new drug and nutraceuticals discovery (Fabricant and Farnsworth, 2001; Lam, 2007; Ngo et al., 2013; Nasri et al., 2014; Lage et al., 2018). For instance, analgesic substances have been derived from natural products (McCurdy and Scully, 2005; Tauchen, 2020), while nutraceuticals with multiple-therapeutic effects have been identified or extracted from natural products (Ghayur et al., 2005; Rahimi Madiseh et al., 2014; Al Hroob et al., 2018). The promising natural products can be identified from traditional Chinese herbal medicine (CHM) (Yuan et al., 2016; Trifković et al., 2019). One well-known example is that the anti-malarial drug artemisinin was discovered from an herb named *qing hao* (*Artemisia annua* L.) based on the clues found in classical Chinese medicine book <*Zhou Hou Bei Ji Fang*>.

In China, CHM is a popular CAM therapy which is reported being prescribed to 60% of migraine patients (Yu et al., 2020). Previous systematic reviews suggested that CHM was effective and safe for migraine management (Zhou et al., 2013; Li et al., 2015; Cai et al., 2018; Lyu et al., 2020), however, the practical value of these research evidence was limited due to considerable heterogeneity in the CHM prescriptions across various studies. Compared to previously published systematic reviews which mainly focused on randomised clinical trials with limited generalisability (Black, 1996), classical Chinese medicine literature contains wider range of invaluable records of treatment experience derived from real-world clinical practice. Identifying commonly used herbs and herb combinations from disease-specific classical literature has been applied as an effective method to select potential candidates for new drug discovery or nutraceuticals (Shergis et al., 2015; Xia et al., 2020). Considering the urgent needs of developing effective migraine therapies, it is important to identify the most promising herbs and herb combinations through systematically reviewing Chinese medicine classical literature.

Therefore, we conducted this data-mining project to collect data relating to herbal prescriptions for the treatment of headache or migraine from Chinese medicine classical literature, and to identify how the key herbs were commonly used as combinations (hereafter namely “core herb combinations”), in order to screen potential candidates for anti-migraine drug discovery. Furthermore, migraine is associated with a group of characteristic symptoms. Clinical management of migraine using CHM takes individual’s symptoms into consideration, therefore it is recognised as a personalised-medicine therapy. In this research, we also aimed to identify herbs which may be beneficial for different migraine-specific symptoms, in order to provide evidence for selecting herbs in clinical practice.

## 2 Materials and methods

Data mining was conducted in the *Encyclopedia of Traditional Chinese Medicine* (*Zhong Hua Yi Dian* 5.0, ZHYD) (Qiu et al., 2014), the most comprehensive digital collection of classical Chinese medicine literature covering over 1,150 books.

### 2.1 Search terms

A list of potential search terms was identified by checking migraine-specific monographs (Wang and Zhang, 2007; Cao and Gao, 2011; Huang and Huan, 2013), clinical expert consensus (Chinese Association of Acupuncture and Moxibustion, 2014), reviews (Ye, 2005; Qin et al., 2010; Wu, 2014; Teng et al., 2017) and theses (Mao, 2013; Fan, 2014). The final search terms used for this research were determined by migraine experts (XL, JS, and SL) through discussion (see Supplementary File S1). These terms were entered into the search box to obtain relevant citations.

### 2.2 Eligible criteria

All citations obtained through searching ZHYD were included unless they met any of the following exclusion criteria:

1) Headaches secondary to eye problems, sinus disorders, toothache, fever, influenza, plague, stroke, brain trauma, tumour and drunkenness;
2) Headaches associated with specific symptoms such as eye redness, eye pain or runny nose that are clinical manifestations of cluster headaches or secondary headaches;
3) Prescribed treatments were not orally administrated herbal medicine;
4) Citations from Materia Medica that explains the effects of herbs for headache or migraine without indicating administration methods;
5) Citations in which migraine or headache was not the main symptom or complaint;
6) Duplicated citations.

### 2.3 Citation screening and scoring

Considering the information recorded in classical literature do not precisely match the descriptions in current medical literature, all eligible citations were scored following pre-determined rules developed according to the diagnostic criteria of “migraine without aura” in the International Classification of Headache Disorders, version 3.0 (ICHD-3) (Olesen, 2018), since this subtype accounts for up to 80% of migraine (Olesen, 2018) (see Supplementary File S1). Citations that scored greater than two were marked as most likely migraine (MLM) citations and included in further analyses.

### 2.4 Data synthesis

The formulae names and herbs from MLM citations were extracted and standardised. Formulae whose names were homonyms (different Chinese characters with the same pronunciation in *pin yi*n [拼音] and similar literal meaning) were standardised according to the most common name. Herb names were standardised based on the Chinese Pharmacopoeia Commission (2020). Citations involving a list of herbs without a formula name were named after a formula with the same reported ingredients, where available, or labelled “no name”. Formulae with the same name that showed diversity in ingredients among different books were distinguished by numbers.

Herbs from the same plant root that were processed in different ways or were named differently over time, were identified as one same herb during frequency analysis. It is worth noting that, herbs *chuan wu*, *cao wu* and *wu tou* are different parts of the same plant and were sometimes prescribed together in the same formula. This was also the case of herbs *sheng jiang* and *gan jiang*, which were the same plant but processed differently. Their original names were kept in formulae ingredients but standardised as one herb in frequency test analyses.

### 2.5 Analytical methods

Data analyses were conducted based on the MLM citations, using SPSS 20 and SPSS modeller 18.0. The frequency and percentage of the formulae and herbs were calculated. Formulae and herbs with high frequency indicate their potential as promising therapies (Wang et al., 2018a).

Association rule based on Apriori algorithm (Agrawal et al., 1993) was applied to identify the core herb combinations. Herbs being cited in more than 8% of the MLM citations are identified as frequent item sets for association rules construction. The Apriori algorithm connects an antecedent item set to a consequent item set based on the notion that these two sets of items co-occur in the database rather than due to a causal effect (Lu et al., 2020).

In the Apriori algorithm, the three standard metrics—support, confidence and lift—were utilised to measure the association between items. Support refers to the prevalence of antecedent item; the higher support levels indicate a higher prevalence of the herb or herb combination. Confidence and lift represent the strength of association, where confidence reflects the possibility of co-occurrences of consequent and antecedent items in the datasets, while lift represents the likelihood of an increase in the consequent given a particular antecedent (Han et al., 2011). The Apriori algorithm has been widely utilised to yield effective herb combinations and discover potential knowledge of Chinese medicine for some diseases (He et al., 2012; Jin et al., 2013; You et al., 2019; Huan et al., 2021).

In addition, a network diagram was generated to illustrate the co-occurrence between individual herbs and visualise the association rules between herbs.

## 3 Results

A total of 13,944 citations were obtained, among which 11,484 citations were excluded for high possibility of being secondary headaches or other primary headache disorders. The remaining 2,460 citations were screened, where 59 duplicates and 1,378 citations that introduced non-oral CHM treatments were excluded. Finally, 614 citations were scored greater than two points and therefore assessed as MLM citations.

**FIGURE 1:**
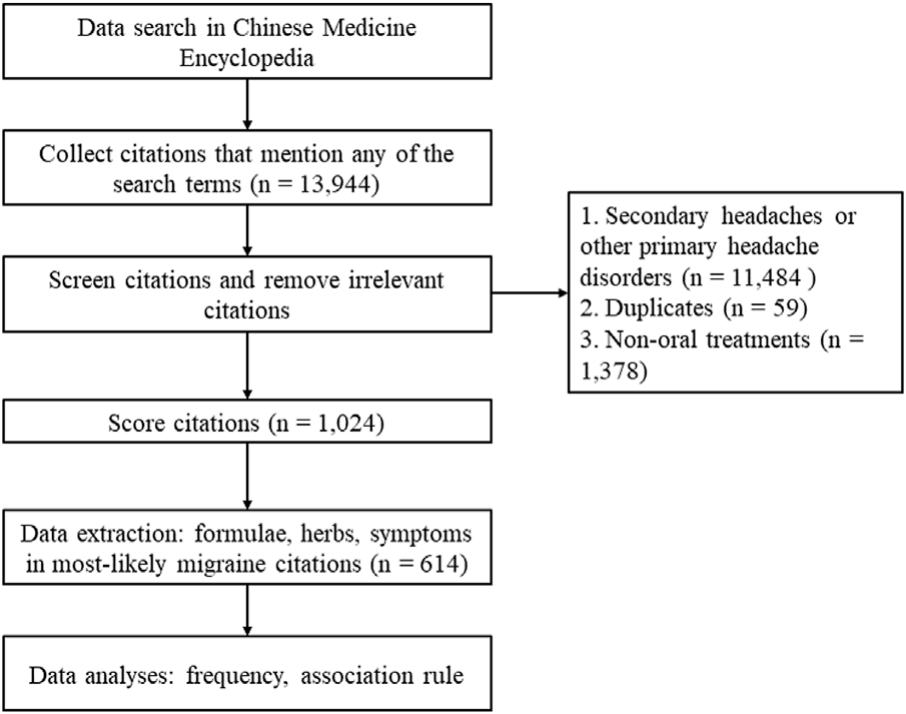
Flow chart of the research procedure.

The research procedure is illustrated in Figure 1.

### 3.1 Frequency analysis

#### 3.1.1 Most frequently used formulae

In the standardised MLM citations, 168 named formulae were collected from 377 citations, formulae from the remaining 237 citations were not named. Formulae being cited more than five times were outlined in Table 1. The formula *chuan xiong cha tiao san* (CXCTS) and its modifications were most popular prescribed for migraine, with a frequency of 37.

**TABLE 1:** Frequently used formulae from the most-likely migraine citations.

Formular name	Frequency	Basic components
*Chuan xiong cha tiao san* and its modified formulae	37	*chuan xiong*, *bai zhi*, *qiang huo*, *xi xin*, *fang feng*, *bo he*, *jing jie*, *gan cao*
*Qing kong gao*	29	*chuan xiong*, *chai hu*, *gan cao*, *huang lian*, *fang feng*, *qiang huo*, *huang qin*
*Cha tiao san*	15	*chuan xiong*, *bai zhi*, *bo he*, *gan cao*, *wu tou (chuan wu)*, *cha*
*Xiong xi wan*	15	*chuan xiong*, *zhu sha*, *shi gao*, *bing pian*, *ren shen*, *fu ling*, *gan cao*, *xi xin*, *xi jiao*, *zi zhi*, *mai dong*, *e jiao*, *cha*, *mi* ^a^, *jiu* ^a^
*Da chuan xiong wan*	15	*chuan xiong*, *tian ma*, *cha*, *mi* ^a^, *jiu* ^a^
*Zhui feng san*	13	*chuan xiong*, *fang feng*, *jing jie*, *jiang chan*, *shi gao*, *bai fu zi*, *tian ma*, *bai zhi*, *qiang huo*, *quan xie*, *di long*, *tian nan xing*, *ru xiang*, *mo yao*, *cao wu*, *chuan wu*, *xiong huang*, *gan cao*, *cha*
*Zhan luo san*	13	*yin su ke*, *chen pi*, *jie geng chai hu*, *deng xin cao*, *gan cao*
*Huang niu nao sui jiu*	12	*chuan xiong*, *bai zhi*, *niu nao*, *jiu* ^a^
*Bai fu zi san*	9	*zao jia*, *bai zhi*, *bai fu zi*, *cha*
*Chuan xiong san (1)*	9	*chuan xiong*, *xi xin*, *qiang huo*, *huai hua*, *xiang fu*, *shi gao*, *jing jie*, *bo he*, *ju hua*, *fang feng*, *yin chen*, *cha*, *gan cao (bai zhi*, *gou teng*, *gao ben)*
*Chuan xiong san (2)*	8	*chuan xiong*, *ju hua*, *shi gao*, *cha*, *jiang chan*
*Chuan xiong shen gong san*	8	*chuan xiong*, *chuan wu*, *bai zhi*, *tian nan xing*, *ma huang*, *gan cao*, *sheng jiang*, *zao*
*Dian tou san*	8	*chuan xiong*, *xiang fu*, *cha*
*Xiong gui tang*	8	*chuan xiong*, *dang gui*
*Xiong xin tang*	8	*chuan xiong*, *fu zi*, *wu tou*, *tian nan xing*, *gan jiang*, *xi xin*, *gan cao*, *sheng jiang*, *cha*
Modified *er chen tang*	8	*chuan xiong*, *chen pi*, *ban xia*, *fu ling*, *gan cao*, *sheng jiang*, *wu mei*, *dang gui*, *chi shao*, *chai hu*, *bai zhi*
*Ru sheng bing zi*	7	*chuan xiong*, *chuan wu*, *tian nan xing*, *gan jiang*, *gan cao*, *tian ma*, *fang feng*, *ban xia*, *xi xin*, *sheng jiang*, *cha*
*Du liang wan*	7	*bai zhi*, *cha*, *mi* ^a^

^a^ *mi* (honey) and *jiu* (yellow wine) are commonly used for preparation of Chinese herbal medicine formulae.

#### 3.1.2 Most frequently used herbs

In total, 293 herbs were prescribed 5,333 times in the MLM citations. The top frequent herbs being cited over 100 times are listed in Table 2. The leading frequently used herb was *chuan xiong*, with a frequency of 357 (59.8%) within the MLM citations. This was followed by herbs *cha* and *gan cao*, which had a frequency of 299 (48.7%) and 279 (45.4%), respectively. The herb *fang feng* was used in 199 (32.4%) citations, and *bai zhi* in 193 (31.4%) formulae. The subsequent frequent herbs were *jing jie* (155, 25.2%), *xi xin* (146, 23.8%), *bo he* (145, 23.6%) and *qiang huo* (142, 23.1%). It is worth noting that the top nine frequently used herbs are also the ingredients of CXCTS*,* which was the most commonly used formula (Table 1).

**TABLE 2:** Frequently used herbs among the most-likely migraine citations.

Herb name in *pin yin*	Frequency (%)	Scientific name^a^
*Chuan xiong*	367 (59.8%)	*Ligusticum striatum* DC.
*Cha*	299 (48.7%)	*Camellia sinensis* (L.) Kuntze
*Gan cao* *^b^*	279 (45.4%)	1. *Glycyrrhiza uralensis* Fisch
		2. *Glycyrrhiza inflata* Batalin
		3. *Glycyrrhiza glabra* L
*Fang feng*	199 (32.4%)	*Saposhnikovia divaricata* (Turcz.) Schischk
*Bai zhi* *^b^*	193 (31.4%)	1. *Angelica dahurica* (Hoffm.) Benth. and Hook.f. ex Franch. and Sav
		2. *Angelica dahurica* var. *formosana* (Boissieu) Yen
*Jing jie*	155 (25.2%)	*Nepeta tenuifolia* Benth
*Xi xin*	146 (23.8%)	1. *Asarum heterotropoides* f. *mandshuricum* (Maxim.) Kitag
		2. *Asarum sieboldii* Miq
*Bo he* *^b^*	145 (23.6%)	*Mentha canadensis* L
*Qiang huo*	142 (23.1%)	1. *Hansenia weberbaueriana* (Fedde ex H.Wolff) Pimenov and Kljuykov
		2. *Hansenia forbesii* (H.Boissieu) Pimenov and Kljuykov
*Wu tou*, including *chuan wu and cao wu*	140 (22.8%)	1. *Aconitum kusnezoffii* Rchb
		2. *Aconitum carmichaelii* Debeaux
*Tian ma* *^b^*	112 (18.2%)	*Gastrodia elata* Blume
*Sheng jiang* *^b^*	110 (17.9%)	*Zingiber officinale* Roscoe
*Shi gao*	109 (17.8%)	Hydrated calcium sulfate

^a^ : Botanical names based on the World Flora Online (WFO) Plant List (https://wfoplantlist.org/ accessed 18 August 2022).

^b^ Dietary medicinal herbs according to the official catalogue of dietary medicinal herbs published by China’s National Health Commission in 2020 (Brower, 1998).

Some of the frequently used herbs for migraine are also dietary medicinal herbs, as defined in the official catalogue published by China National Health Commission (2020), (China National Health and Family Planning Commission, 2018). These herbs include *gan cao* (liquorice), *bai zhi*, *bo he* (peppermint), *tian ma*, *sheng jiang* (ginger). In addition, the second frequently used herb in MLM citations, *cha* (tea) is also used as a common beverage.

### 3.2 Association rules construction of most likely citation herbs

#### 3.2.1 Association rules among all herbs

The Apriori algorithm was utilised to identify the core herb combinations. Support was set as 10%, and confidence level 95%. A total of 61 combinations were obtained (Table 3). The main herbs involved in the combinations include *chuan xiong*, *fang feng*, *qiang huo*, *gan cao*, *cha*, *jing jie*, *xi xin*, *bai zhi* and *bo he.* The association (*cha*, *gan cao*) => (*chuan xiong*) was of the highest degree of support at 26.87%, with a confidence level of 96.36% and a lift of 1.6. Four herb combinations obtained 100% confidence, these are (*qiang huo*, *fang feng*, *cha*) => (*chuan xiong*), (*qiang huo*, *cha*, *gan cao*) => (*chuan xiong*), (*qiang huo*, *fang feng*, *gan cao*, *cha*) => (*chuan xiong*), and (*qiang huo*, *fang feng*, *jing jie*, *cha*) => (*chuan xiong*). The herb combination (*qiang huo*, *gan cao*) => (*fang feng*) had the highest degree of lift at 3.06, with a confidence of 99.19% and support level of 20.68%.

**TABLE 3:** Core herb combinations among the most-likely migraine citations.

Consequent	Antecedent	Support %	Confidence %	Lift
*chuan xiong*	*cha* and *gan cao*	26.87	96.36	1.61
*chuan xiong*	*fang feng* and *cha* and *gan cao*	17.59	99.07	1.66
*chuan xiong*	*jing jie* and *gan cao*	16.29	95.00	1.59
*chuan xiong*	*jing jie* and *fang feng* and *gan cao*	15.47	97.89	1.64
*chuan xiong*	*qiang huo* and *cha*	14.82	98.90	1.65
*chuan xiong*	*qiang huo* and *fang feng* and *cha*	14.33	100.00	1.67
*chuan xiong*	*qiang huo* and *cha* and *gan cao*	14.01	100.00	1.67
*chuan xiong*	*xi xin* and *cha* and *gan cao*	14.01	98.84	1.65
*chuan xiong*	*qiang huo* and *fang feng* and *cha* and *gan cao*	13.84	100.00	1.67
*chuan xiong*	*jing jie* and *qiang huo*	13.52	96.39	1.61
*chuan xiong*	*jing jie* and *qiang huo* and *fang feng*	13.03	97.50	1.63
*chuan xiong*	*bai zhi* and *cha* and *gan cao*	13.03	97.50	1.63
*chuan xiong*	*jing jie* and *fang feng* and *cha*	12.87	98.73	1.65
*chuan xiong*	*jing jie* and *qiang huo* and *gan cao*	12.54	98.70	1.65
*chuan xiong*	*jing jie* and *cha* and *gan cao*	12.54	98.70	1.65
*chuan xiong*	*jing jie* and *qiang huo* and *fang feng* and *gan cao*	12.38	98.68	1.65
*chuan xiong*	*jing jie* and *fang feng* and *cha* and *gan cao*	12.21	98.67	1.65
*chuan xiong*	*xi xin* and *fang feng* and *gan cao*	12.21	96.00	1.61
*chuan xiong*	*bo he* and *cha* and *gan cao*	11.40	98.57	1.65
*chuan xiong*	*bo he* and *fang feng*	11.24	98.55	1.65
*chuan xiong*	*bo he* and *fang feng* and *gan cao*	10.75	98.48	1.65
*chuan xiong*	*bo he* and *bai zhi*	10.75	96.97	1.62
*chuan xiong*	*bo he* and *qiang huo*	10.59	96.92	1.62
*chuan xiong*	*jing jie* and *qiang huo* and *cha*	10.42	100.00	1.67
*chuan xiong*	*jing jie* and *qiang huo* and *fang feng* and *cha*	10.10	100.00	1.67
*fang feng*	*qiang huo* and *chuan xiong*	20.68	95.28	2.94
*fang feng*	*qiang huo* and *gan cao*	20.03	99.19	3.06
*fang feng*	*qiang huo* and *gan cao* and *chuan xiong*	18.73	99.13	3.06
*fang feng*	*jing jie* and *gan cao*	16.29	95.00	2.93
*fang feng*	*jing jie* and *gan cao* and *chuan xiong*	15.47	97.89	3.02
*fang feng*	*qiang huo* and *cha*	14.82	96.70	2.98
*fang feng*	*qiang huo* and *cha* and *chuan xiong*	14.66	97.78	3.02
*fang feng*	*qiang huo* and *cha* and *gan cao*	14.01	98.84	3.05
*fang feng*	*qiang huo* and *cha* and *gan cao* and *chuan xiong*	14.01	98.84	3.05
*fang feng*	*jing jie* and *qiang huo*	13.52	96.39	2.97
*fang feng*	*jing jie* and *qiang huo* and *chuan xiong*	13.03	97.50	3.01
*fang feng*	*jing jie* and *qiang huo* and *gan cao*	12.54	98.70	3.05
*fang feng*	*jing jie* and *cha* and *gan cao*	12.54	97.40	3.01
*fang feng*	*jing jie* and *qiang huo* and *gan cao* and *chuan xiong*	12.38	98.68	3.04
*fang feng*	*jing jie* and *cha* and *gan cao* and *chuan xiong*	12.38	97.37	3.00
*fang feng*	*jing jie* and *bai zhi* and *chuan xiong*	10.75	96.97	2.99
*fang feng*	*qiang huo* and *xi xin*	10.59	96.92	2.99
*fang feng*	*qiang huo* and *bai zhi* and *chuan xiong*	10.59	95.38	2.94
*fang feng*	*jing jie* and *qiang huo* and *cha*	10.42	96.88	2.99
*fang feng*	*jing jie* and *qiang huo* and *cha* and *chuan xiong*	10.42	96.88	2.99
*fang feng*	*bo he* and *qiang huo* and *chuan xiong*	10.26	95.24	2.94
*fang feng*	*qiang huo* and *bai zhi* and *gan cao*	10.10	98.39	3.04
*gan cao*	*qiang huo* and *cha* and *chuan xiong*	14.66	95.56	2.10
*gan cao*	*qiang huo* and *fang feng* and *cha*	14.33	96.59	2.13
*gan cao*	*qiang huo* and *fang feng* and *cha* and *chuan xiong*	14.33	96.59	2.13
*gan cao*	*jing jie* and *qiang huo* and *fang feng*	13.03	95.00	2.09
*gan cao*	*jing jie* and *qiang huo* and *chuan xiong*	13.03	95.00	2.09
*gan cao*	*jing jie* and *qiang huo* and *fang feng* and *chuan xiong*	12.70	96.15	2.12
*gan cao*	*bo he* and *cha* and *chuan xiong*	11.73	95.83	2.11
*gan cao*	*bo he* and *fang feng*	11.24	95.65	2.11
*gan cao*	*bo he* and *fang feng* and *chuan xiong*	11.07	95.59	2.10
*gan cao*	*jing jie* and *qiang huo* and *cha*	10.42	95.31	2.10
*gan cao*	*jing jie* and *qiang huo* and *cha* and *chuan xiong*	10.42	95.31	2.10
*gan cao*	*bo he* and *qiang huo* and *chuan xiong*	10.26	96.83	2.13
*gan cao*	*jing jie* and *qiang huo* and *fang feng* and *cha*	10.10	96.77	2.13
*gan cao*	*jing jie* and *qiang huo* and *fang feng* and *cha* and *chuan xiong*	10.10	96.77	2.13

**FIGURE 2:**
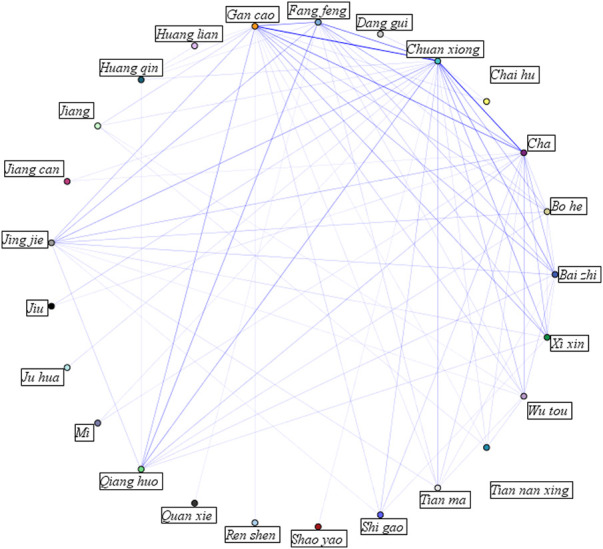
Network diagram of association rules between herbs.

The strengths of association between herbs were measured by the size of the links in the network diagram (Figure 2). The top frequent herb *chuan xiong* was strongly linked with herbs of *gan cao*, *cha*, *fang feng*, *bai zhi*, *qiang huo*, *xi xin*, *jing jie*, *wu tou*, *tian ma* and *shi gao*.

#### 3.2.2 Association rules between symptoms and herbs

The relationships between migraine symptoms and herbs were also explored. The symptoms were taken as antecedent item sets, herbs as consequent item sets. The support level was set as ≥ 5%, and the confidence level was set as ≥ 50%. As shown in Table 4, both symptoms of chronic duration and unilateral headache were closely related to herbs of *chuan xiong*, *gan cao*, *fang feng*, *qiang huo* and *cha*. Particularly, these symptoms were related with the herb *qiang huo* at a higher lift at 2.59. Regarding symptoms of vomiting and nausea, they were specifically related to herbs of *sheng jiang* and *ban xia*, with a particularly high degree of lift over three.

**TABLE 4:** Associations between symptoms and herbs.

Consequent	Antecedent	Support %	Confidence %	Lift
*chuan xiong*	symptom of chronic duration and symptom of unilateral headache	8.14	74.00	1.23
*gan cao*	symptom of chronic duration and symptom of unilateral headache	8.14	68.00	1.50
*fang feng*	symptom of chronic duration and symptom of unilateral headache	8.14	62.00	1.91
*cha*	symptom of chronic duration and symptom of unilateral headache	8.14	62.00	1.27
*qiang huo*	symptom of chronic duration and symptom of unilateral headache	8.14	60.00	2.59
*chuan xiong*	symptom of chronic duration	9.93	62.29	1.04
*cha*	symptom of chronic duration	9.93	59.01	1.21
*fang feng*	symptom of chronic duration	9.93	55.74	1.72
*gan cao*	symptom of chronic duration	9.93	55.74	1.23
*chuan xiong*	symptom of unilateral headache	98.20	60.69	1.02
*sheng jiang*	symptom of unilateral headache and symptom of nausea and vomiting	6.02	59.45	3.32
*ban xia*	symptom of unilateral headache and symptom of nausea and vomiting	6.02	51.35	4.15
*sheng jiang*	symptom of nausea and vomiting	7.32	57.78	3.22
*ban xia*	symptom of nausea and vomiting	7.32	51.11	4.13

## 4 Discussion

### 4.1 Summary of results

Compared with previous data mining studies on classical literature for migraine (Mao, 2013; Wang, 2017; Wang et al., 2020a; Wang and Jiang, 2021), this study adapted the modern diagnostic criteria of migraine as scoring rules to ensure the relevance of migraine-specific citations. In addition, the search terms used in this research covered a broader range, and the *Encyclopedia of Traditional Chinese Medicine* provided a larger collection of classical literature. Consequently, the results based on these citations are considered to be more reliable and relevant than previous studies.

This classical literature-based study revealed that, the most frequently used herb for migraine is *chuan xiong*. The high-frequency usage of *chuan xiong* for migraine is consistent with previous studies, both classical literature (Fan, 2014) and modern literature (Wang et al., 2020a; Lyu et al., 2020; Wang and Jiang, 2021).

The most frequently used formula for migraine in classical literature is CXCTS. Close association rules were constructed, and strong links were examined among the herb ingredients of this formula. This formula is recommended for migraine in the latest Chinese medicine guideline (Ren et al., 2020) and frequently used in contemporary clinical practice, according to a previous survey in Taiwan, China (Chang et al., 2014). Clinical evidence of the effectiveness of formula CXCTS for migraine was systematically analysed by previous systematic reviews (Li et al., 2015; Wang et al., 2019a).

Food and herbal supplements are increasing in popularity for migraine worldwide (Rajapakse and Pringsheim, 2016). The dietary medicinal herb, which is defined as “food, or parts of food, that provide medicinal or health benefits, including the prevention and treatment of disease” (Brower, 1998), can be used to treat or prevent migraine attacks in routine management as supplements and nutraceuticals (China National Medical Products Administration, 2002). The frequently used dietary medicinal herbs identified in this study involve *gan cao*, *bai zhi*, *bo he*, *cha*, *tian ma* and *sheng jiang*. Clinical and experimental evidence supporting the effects of these herbs for migraine is summarised in the following section.

During the association rule construction on migraine symptoms and herbs, it is enlightening to note that symptoms of vomiting and nausea are closely associated with herbs of *sheng jiang* and *ban xia*. These two herbs are commonly paired in Chinese herbal medicine formulae such as *xiao ban xia tang*, and present an enhanced concentration of antiemetic compound (Liu et al., 2015). Therefore, these two herbs can be encouraged to be prescribed for migraine patients suffering from vomiting and nausea. In addition, symptoms of chronic duration and unilateral headache were particularly associated with the herb *qiang huo* at a higher lift. These association rules between specific symptom and herbs may assist individually tailored treatment in clinical practice.

It is commonly known that, some Western traditional medicine herbs, such as butterbur (*Petasites hybridus*), feverfew (*Tanacetum parthenium* L.) and rosemary (*Rosmarinus officinalis* L.) are evidenced beneficial for migraine (Ghasemzadeh Rahbardar and Hosseinzadeh, 2020; Hiemori-Kondo, 2020; Darbahani et al., 2022; Kulinowski et al., 2022). However, they were not identified by this Chinese medicine classical literature data-mining research. This could be explained by their geographical distribution: butterbur is native to Alaska, the contiguous United States, and Canada; feverfew is native to Eurasia, specifically the Balkan Peninsula, Anatolia, and the Caucasus; and rosemary is indigenous to the Mediterranean region. Therefore, they were not used as Chinese herbal medicine in history.

Hepatotoxicity is a concern of CHM which has attracted worldwide attention. Drug-metabolizing enzymes mediating reactive metabolites are one of the most important routes to liver injury. Chinese medicine herbs contain a large number of ingredients that may lead to potentially herb-induced liver injury. For example, *bu gu zhi* (psoralen), *he shou wu* (*Polygonum multiflorum* Thunb), and *lei gong teng* (*Tripterygium wilfordii*) have been reported associated with herb-induced liver injury. The most frequently used Chinese medicine herbs for migraine identified in this study are not necessarily associated with hepatotoxicity, although some live-injury cases were reported. Specifically, *gan cao* and *bo he* were reported to induce liver injuries for patients with chronic hepatitis B infection (Yuen et al., 2006). However, the re-exposure test results, which were reassessed using an established and strict criterion, showed a negative causality for these two herbs (Teschke et al., 2015). *Cha*-associated hepatotoxicity was also once reported (Percevault et al., 2021), but on the other hand, evidence was found to support the hepatoprotective effects of its active compounds, L-theanine (Wang et al., 2020b; Wang et al., 2020c; Zhang et al., 2021; Chen et al., 2022a; Hamad Shareef et al., 2022; Wu et al., 2022). Moreover, *chuan xiong* and *sheng jiang* were reported to exhibit hepatoprotective effects (Lu et al., 2015; Amin et al., 2021; Yaghubi Beklar et al., 2021; Bekkouch et al., 2022; Guo et al., 2022). Even though, additional cautions should be paid regarding herb-hepatotoxicity when prescribing CHM.

### 4.2 Action of herbs

The anti-migraine effects and mechanisms of actions of the formula CXCTS and its ingredients are supported by a number of clinical and pre-clinical research.

The CHM formula CXCTS was proven more effective for migraine compared to flunarizine, as monotherapy or adjunct treatment, by clinical studies, and its anti-migraine effects was achieved by elevating plasma level of 5-hydroxytryptamine (5-HT) and β-endorphin, as well as down-regulating CGRP, substance P and endothelin-1 in the plasma (Yuan and Tan, 2012; Sun and Xu, 2016; Zhou, 2022). These substances are crucial in the aetiology mechanism of migraine attacks (Lassen et al., 2002; Gasparini et al., 2017). A rat-model experiment showed that oral CXCTS (0.12 g/kg) presented anti-migraine effects by regulating the plasma levels of β-endorphin and CGRP when compared to normal saline (Li et al., 2020a). A network pharmacology analysis further confirmed the anti-migraine effects of CXCTS were associated with regulating inflammatory response, vasoconstriction, endocrine-neurotransmitter metabolism through PI3K-AKT, HIF-1 and endocrine resistance signalling pathway (Li et al., 2020b).

The ingredients of CXCTS have also been proven carrying certain actions against migraine. *Chuan xiong* has been traditionally used to alleviate headache in Chinese medicine (Chinese Pharmacopoeia Commission, 2020). The main active compound of *chuan xiong*, Senkyunolide I (orally administered at 16 and 32 mg/kg), presented an analgesics effect by interfering monoamine neurotransmitters and the corresponding turnover rates, as well as nitric oxide (NO), compared with the saline-administered controls in mice models (Wang et al., 2011). Another compound, Ligustrazine (40mg, intravenously injected), showed anti-migraine potentials through inhibiting the c-fos/ERK signalling pathway in the trigeminal ganglion nerve of nitroglycerin-induced migraine rat models (Li et al., 2021). *Bai zhi* is another well-known anti-headache herb in Chinese medicine (Chinese Pharmacopoeia Commission, 2020). The extraction of essential oil of *bai zhi* (orally administered at 35, 70, and 140 mg/kg), ameliorated nitroglycerin-induced migraine rats likely by modulating vasoactive substances, including serum and brain NO levels, plasma CGRP and endothelin levels (Sun et al., 2017). It is worth mentioning that, the combination of *chuan xiong* and *bai zhi* is a classical formula namely *du liang wan*. This herb combination (orally administered at 0.44, 1.31 and 3.93 g/kg) showed anti-migraine effects by adjusting the level of neurotransmitters (5-HT, norepinephrine and dopamine and vasoactive substances (NF-kappaB, nuclear c-fos, inducible nitric oxide synthase, interleukin-1β, and cyclooxygenase-2 levels), consequently relieving neurogenic inflammation (Hou et al., 2017). Additionally, levels of the active compounds of *chuan xiong* (ligustilide, dl-3-n-butylphthalide and senkyunolide A) were significantly evaluated after combining with *bai zhi*, according to a pharmacokinetic comparison research in rat model using a gas chromatography–mass spectroscopy method (Wang et al., 2019b).

The herb *cha* is a daily beverage as well as a medicinal herb with anti-inflammatory activity (Yang et al., 2014). Its major amino acid components, L-theanine, exhibited neuroprotective properties in rodent animals against injury from constriction, stress and toxicity (Jamwal et al., 2017; Jamwal and Kumar, 2017; Unno et al., 2020; Chen et al., 2022b). Additionally, its major polyphenol, epigallocatechin-3-gallate was proved to be beneficial for neuropathic pain alleviation in chronic constriction injured mice models (Bosch-Mola et al., 2017). As a traditional harmonious ingredient, *gan cao* is also a dietary herb. It demonstrated anti-inflammatory through regulating NF-kappaB pathways and tumour necrosis factor for beta-amyloid induced Alzheimer’s disease mice models *in vitro* and *in vivo* (Zhao et al., 2013) and also exerted neuroprotective effects *via* protecting blood brain barrier permeability in lithium-pilocarpine-induced rats (Li et al., 2019). In addition, an active compound of *gan cao* (glycyrrhizic acid) was found antidepressant effects in mild stress mice models (Wang et al., 2018b), that could be beneficial for migraine since depression is a common comorbidity (Buse et al., 2020). *Bo he* is another medicinal herb used as daily beverage. Intranasal application of peppermint oil and cutaneous application of 10% menthol was efficacious and safe as an analgesic for migraine in clinical trials (Borhani Haghighi et al., 2010; Rafieian-Kopaei et al., 2019). However, evidence of effects and safety of orally administered *bo he* for migraine needs further examination.

The herbs *jing jie*, *fang feng* and *qiang huo* are traditionally used to expel Wind and cure cold-induced headache in pairs (Chinese Pharmacopoeia Commission, 2020). However, subcutaneous injections of prim-o-glucosylcimifugin, a molecule from *fang feng*, produced potent anti-nociception by downregulating spinal cyclooxygenase-2 expression *in vivo* and *in vitro* (Wu et al., 2016). Water extract of *jing jie* showed immunoregulation effects by suppression of NF-kappaB in anti-CD3-stimulated mice, which also plays a role in migraine pathology (Kang et al., 2010). As for *qiang huo*, one of its compounds, Notopterol, was evidenced to show analgesic potential (Okuyama et al., 1993; Azietaku et al., 2017). In addition, *qiang huo* formulae were effective for migraine in clinical trials (Ma, 2008; He and Zhang, 2016).

*Xi xin* is a natural analgesic, since its methanol and water extracts exerted anti-nociceptive by activating opioid receptor (Kim et al., 2003; Nie et al., 2021). Safety issue regarding nephrotoxicity is the major concern when using *xi xin* as medicine*.* However, it was evidenced that consumption of *xi xin* with controlled doses and duration was relatively safe (Liu et al., 2021).

Actions of other dietary medicinal herbs are also summarised. Particularly, herb *sheng jiang* was effective for migraine in acute pain abortion with limited side effects, according to previous clinical trials (Cady et al., 2011; Maghbooli et al., 2014). One *in vitro* study attributed these effects to its potential in preventing CGRP release (Slavin et al., 2016). The herb *sheng jiang* also presented anti-nausea effects in clinical practice (Firouzbakht et al., 2014; Mandal et al., 2014). The dual function of anti-migraine and anti-nausea of *sheng jiang* can be informative for migraine accompanied with nausea. *Tian ma* is a common medicinal dietary herb for headache or dizziness (Chinese Pharmacopoeia Commission, 2020). It is usually paired with *bai zhi* in CHM prescriptions*.* The main active compounds of the herb pair, essential oil of *Angelicae dahuricae* radix and Gastrodin were proven to modulate the serum and brain NO levels, decrease plasma CGRP, and increase endothelin levels in nitroglycerine-induced migraine rats (Wang et al., 2016; Sun et al., 2017). When combined with the herb *chuan xiong*, the combination demonstrated analgesics effects in laboratory experiments (Wang et al., 2013; Hu et al., 2016; Vong et al., 2022).

### 4.3 Limitations

This data-mining research is based on Chinese medicine classical literature. Certain limitations should be noted: 1) Migraine was not a clearly defined disease in the pre-modern Chinese medicine literature. It was not feasible to directedly find information related to migraine from the classical literature. Therefore, we selected citations containing symptoms consistent with the diagnostic criteria of migraine according to the ICHD-3. 2) The treatment effects of CHM were not detailed in the classical literature, but it is uncommon that clinicians recorded any ineffective therapies in the books that summarised their clinical experiences. Hence, we consider that the classical literature is valuable in providing potentially effective herbs although rigorous clinical assessment was lacking.

## 5 Conclusion

This study found that the herbal formula CXCTS has been frequently used historically for the treatment of headaches similar to migraine and remains active in contemporary clinical practice. Anti-migraine actions of its herb ingredients strengthen its value in clinical practice and drug-discovery. For migraine associated with nausea and/or vomiting, the herb combination *sheng jiang* and *ban xia* can be prescribed. Moreover, dietary medicinal herbs such as *sheng jiang*, *bo he*, *cha*, *bai zhi*, *tian ma,* and *gan cao*, have the potentials to be further evaluated as nutraceuticals for migraine in self-management.

## Data Availability

The original contributions presented in the study are included in the article/Supplementary Material; further inquiries can be directed to the corresponding authors.

## Abbreviations

CAM, complementary and alternative medicine; CGRP, calcitonin gene-related peptide; CHM, Chinese herbal medicine; CXCTS, *chuan xiong cha tiao san*; MLM, most-likely migraine; NO, nitric oxide; 5-HT, 5-hydroxytryptamine.
