# Supplementary material for: Natural products for migraine: Data-mining analyses of Chinese Medicine classical literature
Source: Front Pharmacol. 2022 Oct 28;13:995559. doi: 10.3389/fphar.2022.995559 (PMC9650126; doi:10.3389/fphar.2022.995559)
Supplement: Supplementary file 1 [file Table1.DOCX]

Supplementary file 1 Search terms and scoring criteria

| Diagnosis criteria from ICHD-3 | Possible descriptions in classical literature | Value |
| --- | --- | --- |
| A. recurrent attacks, more than 5 times. | Words that meant “recurrent”: 复发/时发时止/作止不常/乍差乍发/发歇不定  Words that meant “chronic”: 深久/远年/年深/数岁不已/久不已/久不瘥/久不愈/数年 | 1 |
| B. 4-72 hours of duration of each migraine attack | N/A | N/A |
| C1. unilateral headache | 头角/额角/半边/半爿/鱼尾/左/右/太阳穴痛/偏/头半/太阳痛 | 2 |
| C2. throbbing pain | 头胀/跳痛 | 1 |
| C3. aggravated by routine activities | N/A | N/A |
| C4. moderate-severe pain | 不可忍/剧痛 | 1 |
| D1. nausea or vomiting | 恶心/呕 | 1 |
| D2. photophobia and/ or phonophobia | 畏光/畏声; 不敢见光/恶闻人声 | 1 |
| Search terms (Chinese *pin yin* name) | Meaning of search term | Value |
| E1. *tou feng* | Chronic and recurrent headache | 2 |
| E2. *nao feng* | Chronic and recurrent headache | 2 |
| E3. *nao tong* | Severe headache | 2 |
| E4. *tou tong* | Common headache | 1 |
| E5. *tou teng* | Common headache | 1 |
| E6. *shou feng* | Headache due to Wind attack after bathing | 1 |

Note: ICHD-3: the International Classification of Headache Disorders, version 3.0; N/A: not applicable.
